# A controlled trial comparing dosimetry and radiation pneumonitis between tomotherapy and IMRT in patients with lung or esophageal cancer

**DOI:** 10.1002/acm2.70537

**Published:** 2026-03-18

**Authors:** Anmei Zhang, Yang Zhang, Jingyun Yang, Lu Chen, Na Wu, Jindong Qian, Hongya Dai, Dingqiang Yang, Lirong Zhao, Liangzhi Zhong, Tianxiang Cui, Fan Yang, Guangpeng Chen, Yixing Gao, Wen Luo, Guanghui Li

**Affiliations:** ^1^ Department of Oncology, Xinqiao Hospital Army Medical University Chongqing China; ^2^ Department of Epidemiology, College of Preventive Medicine Army Medical University Chongqing China

**Keywords:** dosimetry, helical tomotherapy, IMRT, radiation pneumonitis, thoracic radiation

## Abstract

**Backgroud:**

The expanding clinical use of helical tomotherapy (HT) has raised concerns regarding its potential to increase low‐dose lung exposure and the risk of radiation pneumonitis (RP) in thoracic radiotherapy. While a few retrospective studies have compared dosimetric parameters and RP rates between HT and fixed‐field intensity‐modulated radiation therapy (IMRT), their findings remain inconsistent, necessitating a prospective randomized controlled trial for clarification.

**Purpose:**

To prospectively compare dosimetric parameters and the incidence of RP between HT and IMRT in patients with lung or esophageal cancer.

**Methods:**

Patients eligible for thoracic radiotherapy were enrolled. Both HT and IMRT plans were designed and optimized for each patient, with a prescription equivalent dose in 2 Gy /fraction (EQD2) ≥50 Gy to the gross tumor volume (GTV). Plans were evaluated based on target dose coverage, dose‐volume histograms, and other dosimetric indices. RP was diagnosed and graded according to the Common Terminology Criteria for Adverse Events (version 5.0). Risk factors for RP were identified using univariate analysis.

**Results:**

Between February and September 2022, 110 consecutive patients with lung or esophageal cancer were enrolled and randomly assigned in a 1:1 ratio to either the HT group (*n* = 54) or the IMRT group (*n* = 56). Compared with IMRT, HT had a significant reduction in lung V20 (*p* = 0.002) and mean lung dose (*p* = 0.013). Furthermore, the HT group exhibited a superior conformity index for the planning gross tumor volume of the primary lesion (PGTVp) (*p* = 0.004) and a lower homogeneity index for all planning target volumes (PTVs) (*p* < 0.001). At a median follow‐up of 14.0 months, the rate of grade≥2 RP for the entire cohort was 14.5%, with no significant differences between the HT and IMRT groups (*p* = 0.61).

**Conclusions:**

Compared with fixed‐field IMRT, HT provided superior dose distribution to the PTVs while maintaining a comparable incidence of RP in patients undergoing thoracic radiotherapy.

## 1 INTRODUCTION

Helical tomotherapy (HT) represents a distinct technological approach compared to conventional fixed‐field intensity‐modulated radiation therapy (IMRT). By integrating a linear accelerators with spiral computed tomography (CT) technology, HT enables precise 360° radiation delivery and optimized dose of distribution to the target volume.^1^, ^2^ The system combines a megavoltage CT imaging unit and a binary pneumatic multi‐leaf collimator with rapid leaf opening times (about 20 ms), offering significant advantages for precision radiotherapy and an extended treatment field along the patient's longitudinal axis. It was primarily employed for treating head‐neck and prostate cancers, especoally for complex and large volume targets, and was also applicable to thoracic malignancies.^3^, ^4^

Radiation pneumonitis (RP) is one of the most common complications in patients undergoing thoracic radiotherapy.^5^, ^6^ Its incidence correlates positively with the volume of normal lung tissue exposed to low‐dose radiation, as reflected in parameters such as V5, V20, and V30.^6^, ^7^ The reported incidence of severe (≥ grade 3) pneumonitis ranged from 2.8% to 7%,^8^, ^9^, ^10^ with associated mortality rates reaching up to 50%, while any grade RP occurred in 5% to 78% of cases.^11^, ^12^ Even asymptomatic RP, often termed subclinical lung injury, can adversely affect patients by reducing lung function reserve and imposing long‐term impacts on cardiopulmonary health. The increasing use of immune‐checkpoint inhibitors in thoracic oncology has raised concerns about the potential for heightened radiation‐induced lung injury. Comparative dosimetric studies have shown that both VMAT and HT tend to increase low‐dose lung irradiation compared to static techniques.^13^, ^14^ This elevated low‐dose exposure may, in turn, amplify the risk of RP, particularly in patients undergoing immunotherapy. These finding have raised concerns among radiation oncologists regarding a potentially elevated risk of RP when using rotational therapy techniques for thoracic radiotherapy.

To date, only limited number of retrospective studies had compared the incidence of RP and related dosimetric parameters between HT and IMRT in patients undergoing thoracic radiation. Retrospective analysis by Meng et al. and Wang et al. indicated that HT did not increase low‐dose radiation to normal lung tissue compared to fixed‐field IMRT in patients with non‐small cell lung cancer (NSCLC) or esophageal cancer.^15^, ^16^ Similarly, a retrospective study by Figlia et al. reported that HT did not elevate the incidence of RP in lung cancer radiotherapy.^17^ In contrast, Kim et al. observed RP incidence rates of 38.7%, 54.8%, and 6.5% for grade 1, 2, and 3, respectively, in lung cancer patients treated with HT. The rate of grade ≥2 RP was slightly higher than that to reported with IMRT in contemporary studies. Moreover, they identified ipsilateral V10 as the strongest predictor of RP.^18^ These conflicting results have influenced the clinical adoption of HT for thoracic malignancies. Given the inconsistent findings from retrospective studies, we conducted a randomized controlled trial—the first to prospectively compare dosimetric parameters and RP incidence between HT and fixed‐field IMRT in patients with thoracic cancer.

## 2 METHODS

### 2.1 Study design and patient eligibility and enrollment

This randomized controlled trial was designed to compare lung toxicity and dosimetric differences between HT and linac‐based fixed‐field IMRT in patients with lung or esophageal cancer recieving thoracic radiotherapy. The study was conducted at Xinqiao Hospital. The protocol was approved by the hospital's ethics committee (No. 2021‐163‐01) and registered with the Chinese Clinical Trial Registry (www.chictr.org.cn. Identifier: ChiCTR2200056025. Registry date: Jan 31, 2022). All the participants provided written informed consent before enrollment.

Eligible patients were aged 18 years or older, had an Eastern Cooperative Oncology Group (ECOG) performance status (PS) of 0–2, and had a histologically confirmed malignant tumor of the esophagus or lung eligible for thoracic radiotherapy, along with adequate hematologic and organ function. Tumor staging was performed according to the 8th edition (2018) of the American Joint Committee on Cancer TNM staging.

### 2.2 Radiation treatment planning and randomization

All patients underwent standard radiotherapy planning, including four‐dimensional CT simulation for motion assessment and target delineation. For each patient, paired IMRT and HT plans were developed for dosimetric comparison. The CT simulation for lung cancer extended from the hyoid bone to the infra‐diaphragmatic regions, while for esophageal cancer, it spanned from the hyoid bone to the celiac trunk. All treatment plans were optimized using the TomoHD™ Planning Station (version 5.1.1.6; Accuray) and Monaco (version 5.11.03; Elekta). For HT planning, the medical physicist delineated a larger directional block region on the contralateral side and a smaller directional block region on the ipsilateral side. Radiation within these regions was blocked to prevent direct exposure to lung tissue, thereby reducing the delivered dose. The optimization process incorporated a maximum dose constraint (≤10 Gy) for each block and limited the V5 to ≤10%. This strategy effectively reduces the volume of lung exposed to low‐dose radiation. The extent of block delineation and optimization parameters, including dose constraints, are typically determined based on the medical physicist's experience. For IMRT planning, 5–7 beams are routinely employed. Beam angles are optimized to account for tumor location, functional lung sparing, and protection of adjacent organs at risk (OARs). Plan optimization applies dose–volume constraints to concentrate high dose within the target while achieving sharp dose fall‐off near OARs, thereby reducing risks of RP, esophagitis, and cardiac toxicity. All treatment plans underwent quality assurance (QA) verification using the ArcCHECK® system. In the QA analysis, global gamma evaluation with a 3%/2 mm criterion and a 10% low‐dose threshold was applied to both HT and IMRT plans, consistent with the recommendations outlined in the American Association of Physicists in Medicine (AAPM) Task Group Report No. 218 for IMRT and VMAT QA. Target volumes, including gross tumor volume of the primary lesion (GTVp), GTV of metastatic lymph nodes (GTVnd), and clinical target volume (CTV), were delineated and expanded following RTOG or EORTC guidelines. Planning target volumes (PTV), namely PGTVp, PGTVnd, and PCTV were generated by applying 5‐mm isotropic margin to the respective GTVp, GTVnd, and CTV.

Both IMRT and HT plans were evaluated according to predefined dose‐volume constraints. The prescribed equivalent dose in 2 Gy/fraction (EQD2) to the tumor was ≥50 Gy, provided it could be safely delivered within the constraints. Patients were eligible for randomization only if both plans met all dose constraints for lung, heart, esophagus, and spinal cord. Eligible participants were randomly assigned (1:1) to receive either HT or IMRT (control). An independent statistician, not involved in the clinical conduct of the trial, performed randomization using a computer‐generated permuted block scheme (block size of 4). The allocation sequence was concealed from both participants and investigators until the interventions were assigned. Due to the nature of the intervention, blinding of participants or study personnel was not feasible. Data were recorded using paper‐based case report forms, and RP events were documented as they occurred.

### 2.3 Plan evaluation

Dose‐volume histograms (DVH) were generated and analyzed to compare plans between the two modalities. OARs included the normal lung, heart, esophagus, and spinal cord for lung cancer, and the lung, heart, and spinal cord for esophageal carcinoma. Evaluated parameters included lung V5, V20, V30, mean lung doses (MLD), heart V30 and V40, mean heart dose (MHD), esophageal V50, and maximum spinal cord dose. Contouring of the heart and lung followed the validated University of Michigan Heart Atlas guidelines. Dose constraints for OARs were specified in the study protocol. During treatment planning, the goal was to maintain lung V20 < 28%, MLD < 1500cGy, heart V40 < 30%, and spinal cord Dmax < 45 Gy, whenever feasible.

### 2.4 Assessment of end points

The primary endpoint of the study was the incidence of RP, graded according to the Common Terminology Criteria for Adverse Events (version 5.0). Diagnosis of RP required the following: radiation exposure involving a significant volume of normal lung tissue; radiographic evidence of inflammatory changes consistent with the radiation dose distribution within 6 months after starting radiotherapy; and symptoms attributable to RP. All patients underwent their first follow‐up 1 month post‐radiotherapy, with subsequent re‐evaluations every 2–3 months. A chest CT scan was routinely performed at each follow‐up, regardless of the presence of clinical symptoms. If patients developed suspected RP‐related symptoms (e.g., dry cough, dyspnea, or fever), they were scheduled for an unscheduled re‐evaluation with a CT scan. An internal outcome review group assessed all RP events to ensure objectivity and consistency in reporting.

The secondary endpoint was the dosimetric comparison between HT and IMRT. Conformity and homogeneity indices were calculated and analyzed for both modalities. The homogeneity index (HI) was defined as (D2%—D98%)/D50%, where D2%, D98%, and D50% represent the doses to 2%, 98%, and 50% of the target volume, respectively.^19^ The conformity index (CI) was defined as (VTref/VT)x(VTref/Vref),^19^ where VTref was the volume of the target receiving a dose equal to or greater than the reference dose, VT was the volume of target, and Vref was the volume receiving a dose equal to or greater than the reference dose (treated volume).^20^ The time of RP occurrence was calculated from the start of radiotherapy to the first diagnosis of RP.

The exploratory endpoint included risk factor analysis for RP in the HT group.

### 2.5 Data analysis

The sample size was determined based on the data reported in the literature. Prior studies reported a grade ≥ 2 RP incidence of approximately 25% with IMRT.^12^, ^21^, ^22^ Assuming 80% power, a type I error rate of 0.05, and a 1:1 randomization, the projected incidence of grade ≥ 2 RP in the control group (IMRT) was 25%, the non‐inferiority margin of the experimental group (HT) was 10%, and the loss to follow‐up rate was 10%. It was estimated that at least 54 patients were required for each study group. Calculation were performed using Power Analysis (version 11.0) and Sample Size software (version 11.0) (Appendix 1).

Statistical analyses were performed using PASS Statistics version 21 (SPSS Inc.). Categorical data were presented as counts and percentages, and continuous data were summarized as means and standard deviations (SDs). Pearson's chi‐square test or Fisher's exact test was used to compare proportions depending on the situation. For continuous variables, the Student's t‐test or Mann–Whitney U‐test was used for parametric and non‐parametric data, respectively, with analysis of variance (ANOVA) on ranks applied for comparisons involving more than two groups. Statistical significance was set at *p* < 0.05.

No Large Language Models were utilized in this study.

## 3 RESULTS

**FIGURE 1:**
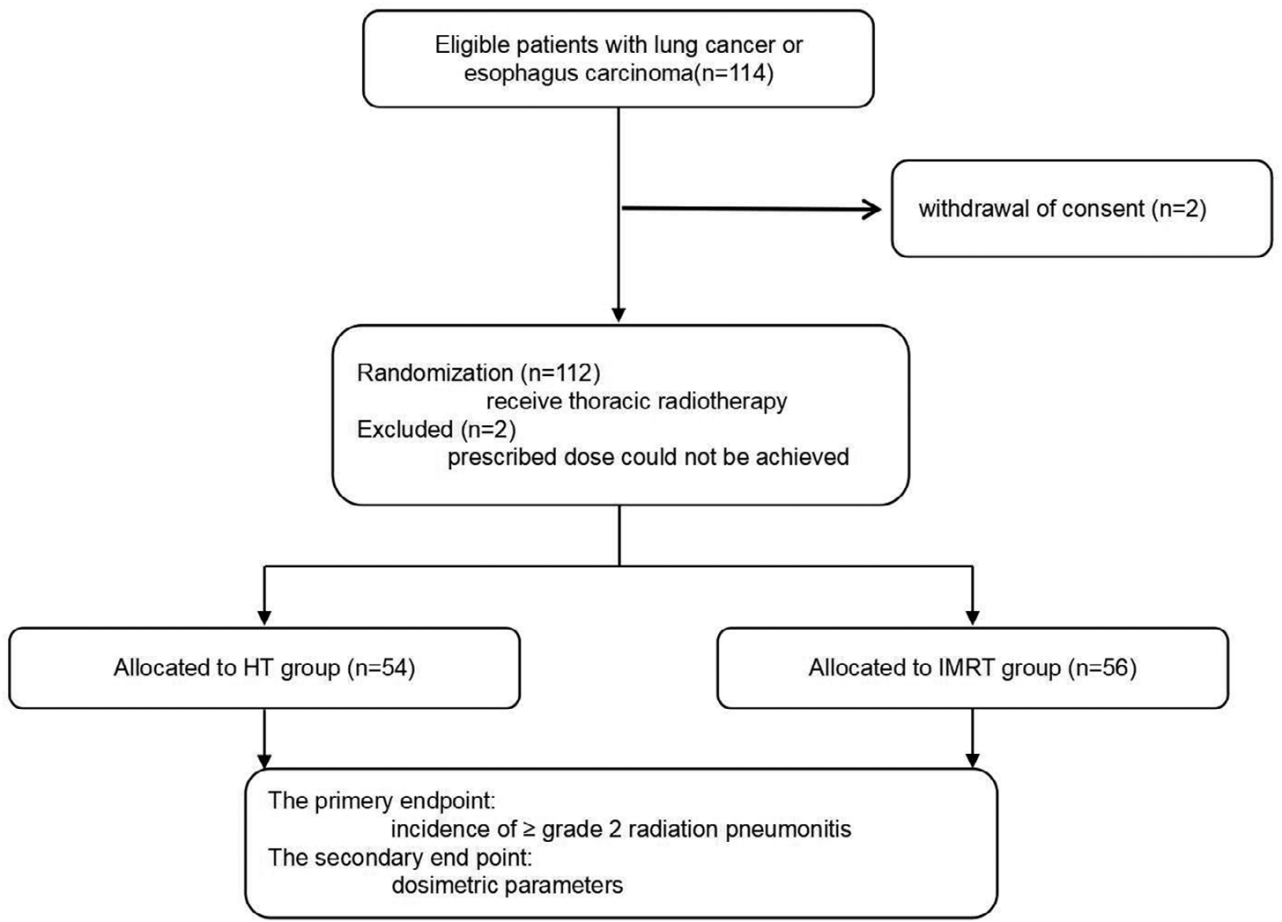
Study profile. HT = helical tomotherapy, IMRT = intensity‐modulated radiation therapy.

Between February 16, 2022, and September 27, 2022, 114 patients consented to participate; two patients were excluded before the planning process began due to withdrawal of consent. A total of 112 enrolled patients completed the planning process, with two plans each; 110 of the 112 patients were randomly assigned and treated accordingly (IMRT [*n* = 56] and HT [*n* = 54], Figure 1). Two patients were not treated with the protocol because the prescribed dose could not be achieved. Baseline characteristics were well balanced between the study groups. Seventeen (15.5%) of the 110 participants were female and 93 (84.5%) were male, and the mean age was 61.3 years (SD 9.2). The mean planning treatment volume of GTV, GTVnd and CTV were 158.9 cm^3^ (SD 160.7), 55.4 cm^3^ (SD 41.7), 272.3 cm^3^ (SD 256.5), respectively (Table 1).

**TABLE 1:** Baseline characteristics.

	IMRT (*n* = 56)	HT (*n* = 54)	*P* value
Age (year)	59.7 (8.5)	63.1 (9.6)	0.06
Sex			0.16
Female	6 (10.7%)	11 (20.4%)	
Male	50 (89.3%)	43 (79.6%)	
Underlying respiratory disorders			0.27
Yes	6 (10.7%)	2 (3.7%)	
No	50 (89.3%)	52 (96.3%)	
Cumulative smoking exposure (in cigarette‐years)			0.19
≥400	35 (62.5%)	27 (50.0%)	
<400	21 (37.5%)	27 (50.0%)	
Histology			
NSCLC	17 (30.4%)	23 (42.6%)	0.18
SCLC	18 (32.1%)	11 (20.4%)	0.16
Esophagus carcinoma	21 (37.5%)	20 (37.0%)	0.96
ECOG			0.27
0‐1	54 (96.4%)	49 (90.7%)	
2	2 (3.6%)	5 (9.3%)	
Hypofractionated radiotherapy			0.46
Yes	16 (28.6%)	19 (35.2%)	
No	40 (71.4%)	35 (64.8%)	
PTV volume(ml)			
PGTVp	162.0 (136.3)	155.5 (183.8)	0.2
PGTVnd	54.63 (44.58)	56.18 (40.57)	0.94
PCTV	306.14 (198.50)	272.36 (130.94)	0.53
Concurrent therapy			0.73
Yes	28 (50.0%)	19 (35.2%)	
No	28 (50.0%)	35 (64.8%)	

*Note*: Data are *n* (%) or mean (SD) unless otherwise stated.

Abbreviations: NSCLC, non‐small cell lung cancer; SCLC, small cell lung cancer; PTV, plannning treatment volume; PGTV*p*, planning gross tumor volume of primary lesion; PGTVnd, planning gross tumor volume of metastatic lymph nodes; PCTV, planning clinical target volume.

### 3.1 Primary end points by RP

The median follow‐up times for the IMRT group were 14.2 months; corresponding median follow‐up times for the HT group were 13.8 months. Sixteen patients developed grade ≥ 2 RP (7 in HT group, while 9 in IMRT group). At six months, the all‐grade RP rates were 72.7% in all patients (IMRT, 75% vs. HT, 70.4%; *p* = 0.586). The results of RP were shown in Table 2. In the HT group, two patients developed grade 4 RP, whereas in the IMRT group, one patient developed grade 3 and one case of grade 4 RP. No cases of grade 5 RP were observed in either group. Grade ≥ 2 RP rates in our cohort were lower than the 25% assumed in the trial design. In both the lung cancer and esophageal cancer subgroups, no statistically significant differences were observed in the incidence of all‐grade RP or grade ≥ 2 RP between the HT and IMRT groups, with all P‐values exceeding 0.5. These findings are consistent with those observed in the overall population (Supplementary Table S1). Furthermore, the median time of occurrence of RP at all levels was 80.5 days in the HT group and 84 days in the IMRT group, respectively, and there was no statistically significant difference between the two groups (*p* = 0.53).

**TABLE 2:** Incidence of RP.

	IMRT (*n* = 56)	HT (*n* = 54)	*P* value
All grade of RP			0.59
Yes	42(75.0%)	38 (70.4%)	
No	14 (25.0%)	16 (29.6%)	
≥ grade 2 RP			0.64
Yes	9 (16.1%)	7 (13.0%)	
No	47 (83.9%)	47 (87.0%)	
Days from onset of RT to RP (Days)^*^	84 (52.2)	80.5 (57.2)	0.21

*Note*: Data are *n* (%) or mean (SD) unless otherwise stated.

Abbreviation: RP, radiation pneumonitis.

* : *n* = 42 in IMRT group, *n* = 38 in HT group.

### 3.2 SECOND end points by dosimetric comparison

#### 3.2.1 Comparison of target dose coverage

For each enrolled patient, one paired plan was designed using HT and IMRT techniques to facilitate dosimetric comparison. The PTV dosimetric parameters and comparisons between the two radiation techniques were summarized in Table 3. Compared with IMRT, HT generally provided a higher CI and lower HI, indicating a more conformal and homogeneous dose distribution to the PTV. The mean CI for PGTVp was significantly higher than that for HT compared to IMRT (0.7 vs 0.6, *p* = 0.004). The mean HIs were also significantly better for the PGTVp, PGTVnd, and PCTV by HT than by IMRT (0.06 vs 0.1, 0.05 vs 0.1, 0.18 vs 0.3 respectively, all *p* < 0.001).

**TABLE 3:** Dose distributions for target volume and OARs.

	IMRT	HT	*P* value
CI			
PGTVp *n* = 108	0.6 (0.1)	0.7 (0.2)	0.004
PGTVnd *n* = 16	0.4 (0.2)	0.5 (0.2)	0.075
PCTV *n* = 40	0.6 (0.1)	0.6 (0.1)	0.894
HI			
PGTVp *n* = 108	0.1 (0.04)	0.06 (0.03)	<0.001
PGTVnd *n* = 16	0.1 (0.03)	0.05 (0.02)	<0.001
PCTV *n* = 40	0.3 (0.07)	0.18 (0.06)	<0.001
Total lung (*n* = 110)			
MLD (Gy)	10.3 (3.1)	9.4 (3.0)	0.013
V5 (%)	44.6(12.3)	41.7 (13.1)	0.063
V20 (%)	19.3 (7.1)	16.6 (6.7)	0.002
V30 (%)	10.5 (5.0)	9.5 (5.0)	0.11
Heart (*n* = 110)			
MHD (Gy)	12.6 (8.4)	12.9 (8.3)	0.727
V30 (%)	15.4 (14.1)	15.4 (12.2)	0.796
V40 (%)	8.3 (8.5)	8.8 (7.8)	0.561
Spinal cord (*n* = 110)			
Dmax (Gy)	36.1 (8.3)	36.0 (8.7)	0.917
Esophagus (*n* = 68)			
V50 (%)	15.6 (14.7)	17.5 (16.7)	0.604

*Note*: Data are *n* (%) or mean (SD) unless otherwise stated.

Abbreviations: CI, conformity index; HI, homogeneity index; MLD, mean lung doses; MHD, mean heart doses; Dmax, maximum dose.

#### 3.2.2 Dosimetric comparison for the OARs

**FIGURE 2:**
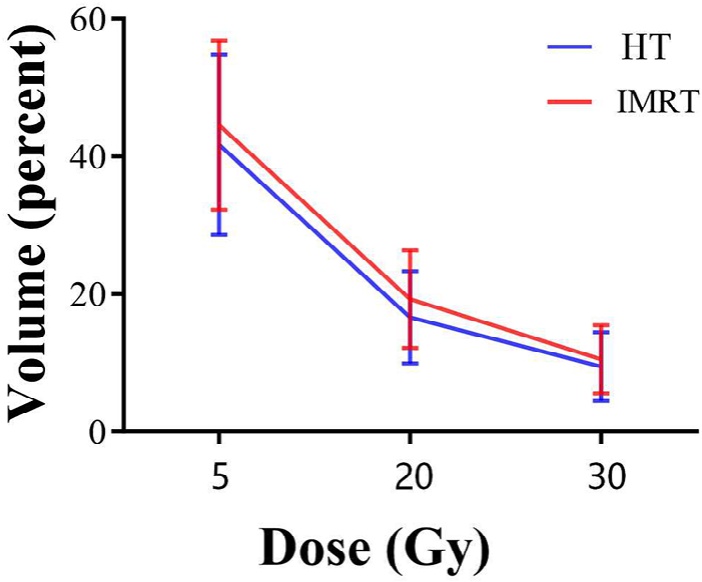
Illustration of the lung volume receiving 5, 20, and 30 Gy with HT (blue line), and IMRT (red line).

**FIGURE 3:**
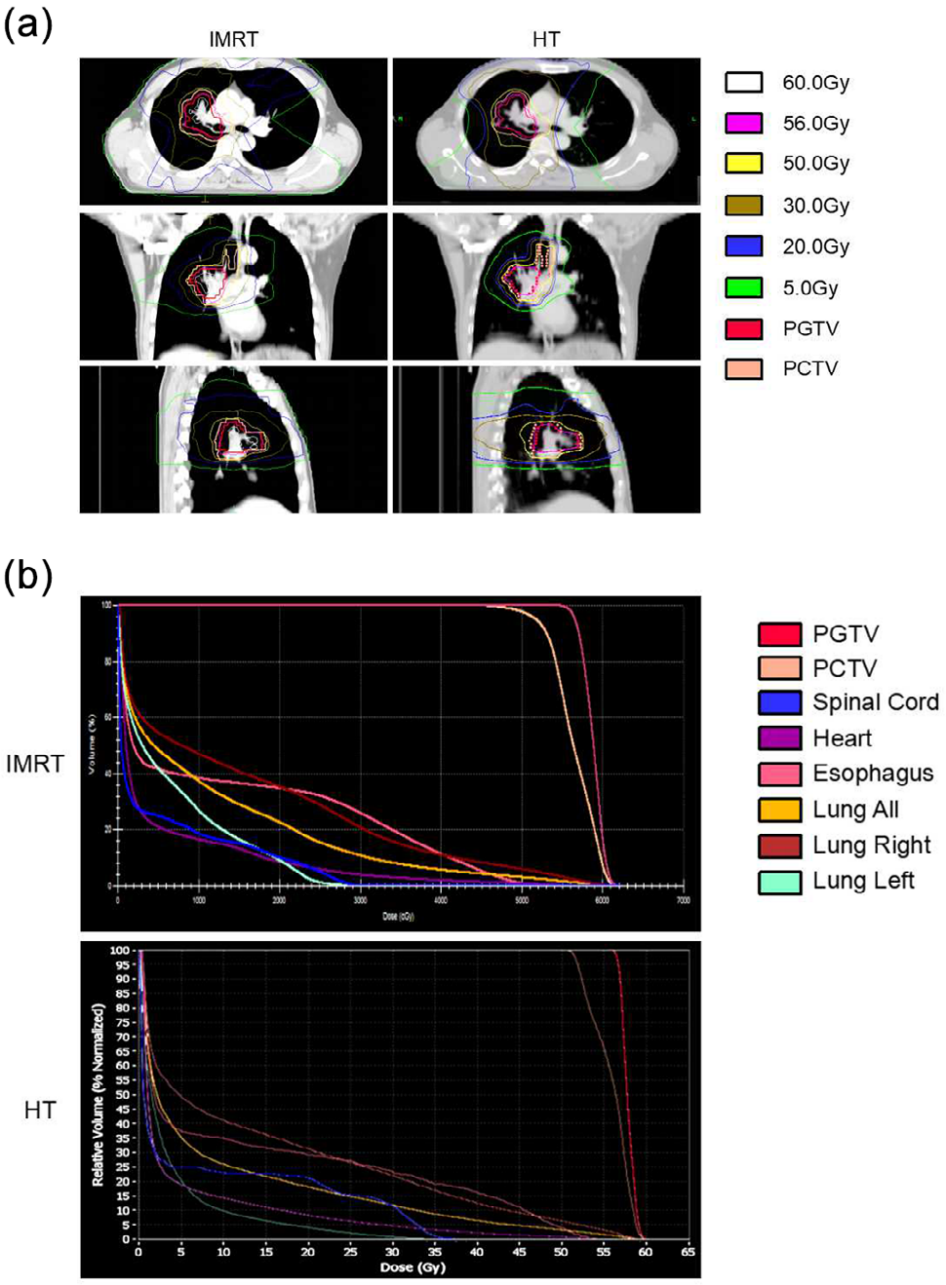
Typical distributions for HT and intensity modulated radiotherapy (IMRT) plans for a patient showing the same CT slice (a). Te PTV for GTV (PGTV) was painted in red. Te PTV for CTV (PCTV) was painted in orange. Te white, pink, yellow, brown, blue and green lines represent the dose curves of 60, 56, 50, 30, 20, and 5 (the prescription dose) Gy, respectively. Dose‐Volume Histogram for HT and intensity modulated radiotherapy (IMRT) plans for the same patient (b). Te PTV for GTV (PGTV) was painted in red. Te PTV for CTV (PCTV) was painted in orange. Te blue, purple, pink, dark orange, dark red and green lines represent the dose curves of spinal cord, heart, esophagus, lung‐all, lung‐right and lung‐left, respectively.

As observed here, the V5, V20, V30, and MLD for the whole lung in HT were all lower than those in the IMRT group (41.7% vs 44.6%, 16.6% vs 19.3%, 9.5% vs 10.5%, 9.4 Gy vs 10.3 Gy, respectively), and the differences in the V20 and MLD for the whole lung were significantly different between the two groups (*p* = 0.002 and *p* = 0.013, respectively) (Table 3 and Figure 2). The V30 (15.4% vs 15.4%), V40 (8.8% vs 8.3%), and MHDs (12.9 Gy vs 12.6 Gy) for the heart; maximum doses to the spinal cord (36.0 Gy vs 36.1 Gy); and V50 for the esophagus (17.5% vs 15.6%) were comparable between the two radiation techniques (*p* > 0.05). Considering the differences in radiation fields and dose distributions between lung cancer and esophageal cancer, we performed subgroup analyses for each cancer type. In the lung cancer subgroup, HT showed trends toward lower V20, a well‐established indicator closely related to the occurrence of RP, along with reduced V5 and MLD compared to IMRT (*p* = 0.06, 0.07, and 0.10, respectively). Similarly, in the esophageal cancer subgroup, V20 was significantly lower in HT plans (*p* = 0.03). These findings align with those observed in the overall population. However, due to the limited sample size for each subgroup analysis, no statistically significant differences were found, except for V20 in the esophageal cancer group. Figure 3 illustrates the typical dose distributions (a) and dose‐volume histograms (DVHs) (b) for the HT and IMRT plans of a representative patient.

#### 3.2.3 Exploratory end points by risk factors for RP in the HT group

To further investigate the risk factors related to grade ≥2 RP in HT therapy, a subgroup analysis was carried out in this study among the patients who underwent HT radiotherapy for chest lesions (Table 4). The findings indicated that the escalation of the count of white blood cells (*p* = 0.021), the count and percentage of neutrophils (*p* = 0.016, *p* = 0.001, respectively), the reduction of the count and percentage of lymphocytes (*p* = 0.005, *p* = 0.005, respectively) within 1 month after radiotherapy, the augmentation of V5, V20, and D_mean_ of both lungs (*p* = 0.018, *p* = 0.021, *p* = 0.01, respectively), as well as the increase of lactic acid within 3 months after radiotherapy (*p* < 0.001) were associated with the occurrence of grade ≥2 RP.

**TABLE 4:** Univariate analyses for HT related RP.

	≥ grade 2 RP (*n* = 7)	grade 0–1 RP (*n* = 47)	*P* value
**Pre‐radiation characteristics**			
Age yrs	61.4 (7.6)	63.3 (10.0)	0.637
Male	7 (100%)	36 (76.6%)	0.322
Underlying pulmonary diseases	0 (0%)	2 (0.04%)	1
Smoking index ≥ 400 cigs/yr	6 (85.7%)	21 (44.7%)	0.1
Hypofractionated radiotherapy	1 (14.3%)	18 (38.3%)	0.4
Immunotherapy within 3 months before radiation	4 (57.1%)	15 (31.9%)	0.379
Concurrent chemotherapy	4 (57.1%)	18 (38.3%)	0.425
**Dosimetric parameters**			
Lung V5(%)	50.7 (5.6)	38.7 (13.3)	0.018
V20(%)	21.0 (4.8)	15.4 (7.4)	0.021
V30(%)	11.5 (3.9)	8.4 (5.0)	0.087
MLD (Gy)	11.2 (1.8)	8.8 (3.2)	0.01
**Hematological parameters after radiation**			
Count of white blood cells^*^(×10^9^/L)	11.5 (4.0)	8.0 (4.3)	0.021
Count of neutrophils^*^(×10^9^/L)	10.3 (3.9)	6.4 (4.2)	0.016
Percentage of neutrophils^*^(%)	92.8 (5.1)	79.6 (9.5)	0.001
Count of lymphocyte^*^(×10^9^/L)	0.2 (0.1)	0.6 (0.4)	0.005
Percentage of lymphocytes^*^(%)	3.6 (2.8)	10.7 (7.4)	0.005
Count of red blood cells^*^(×10^12^/L)	3.0 (0.5)	3.5 (0.7)	0.072
Count of hemoglobin^*^(g/L)	94.6 (17.1)	109.2 (21.1)	0.072
Count of HCT^*^(%)	28.8 (5.0)	33.1 (6.2)	0.071
Count of platelets^*^(×10^9^/L)	190.6 (28.8)	192.9 (75.9)	0.883
Count of lactic^**^(mmol/L)	6.5	1.15 (0.4)	<0.001

*Note*: Data are *n* (%) or mean (SD) unless otherwise stated.

* : *n* = 7 in ≥ grade 2 RP group, *n* = 44 in gradde 0–1 RP group.

** : *n* = 1 in ≥ grade 2 RP group, *n* = 6 in gradde 0–1 RP group.

## 4 DISCUSSION

To our knowledge, this represents the first prospective randomized clinical trial designed specifically to compare the incidence of RP and perform a comprehensive dosimetric evaluation between HT with IMRT for thoracic malignancies. Among all 110 patients, 14.5% (16/110) developed grade ≥ 2 RP. Through this conducted randomized controlled trial, we observed no significant difference in the incidence of RP between HT and fixed‐field IMRT. The overall incidence of all‐grade RP in our study was 72.7%, which aligns with the findings from previous studies.^23^, ^24^ The relatively high detection rate may primarily be attributed to our routine CT follow‐up strategy, which allowed for the identification of asymptomatic grade 1 RP, characterized solely by radiological changes. The incidence of grade ≥ 2 pneumonia in patients who received IMRT was 16.1%, whereas that in patients who received HT was 13.0%. This study indicates that no statistically significant difference in the incidence of grade ≥ 2 RP was observed between fixed‐field IMRT and HT in lung and esophageal cancer patients undergoing thoracic radiotherapy. Of course, the rate of grade ≥ 2 RP in this study was marginally lower than those reported in previous studies,^25^, ^26^, ^27^ which probably because of strict control of the dose to the normal lung tissue during the planning design and careful follow‐up and management of patients.

In terms of target dose conformity and homogeneity indices, HT demonstrated superior dose modulation capability compared with fixed‐field IMRT. Comparative analysis of 110 matched treatment plans for lung and esophageal cancer patients revealed that HT achieved significantly higher dose conformity indices for PGTVp and significantly better dose homogeneity indices for PGTVp, PGTVnd, and PCTV than fixed‐field IMRT. The results of CI and HI for HT group in this study were consistent with the data results reported by Klunklin et al.^13^ and Xu et al.^14^ It is worth noting that he CI value of the IMRT group was lower than those reported in prior studies. Xu et al.^14^ reported a CI value of 0.75 for the IMRT group, and Li et al.^28^ reported a CI value of 0.69. This discrepancy may be attributed to our institution's stringent dose constraints for OARs, particularly the lung, where we aimed to keep V20 < 28% and MLD < 15 Gy, potentially at the cost of some target conformity in the IMRT plans.

Previous studies have not reached a consensus on whether HT expands low‐dose irradiation of normal lung tissue.^15^, ^16^ In this study, we concurrently devised IMRT and HT plans for each enrolled patient to facilitate the comparison of dosimetric parameters. Results demonstrated that while significantly improving target dose conformity and homogeneity, HT did not increase doses to OARs. Notably, the HT group showed significantly lower lung V20 (*p* = 0.002) and mean lung dose (MLD; *p* = 0.013) compared to fixed‐field IMRT. This lung‐sparing advantage was particularly pronounced in cases with multiple lesions separated by large spans. Our data conclusively demonstrate that HT did not expand low‐dose lung irradiation volumes relative to fixed‐field IMRT, addressing a key clinical uncertainty. Our results is similar to prior retrospective studies supporting the advantages of VMAT over IMRT.^13^, ^28^ However, prospective head‐to‐head comparisons between HT and VMAT remain limited, particularly with respect to clinical endpoints like RP. Given the growing role of VMAT in clinical practice, future studies that directly compare HT and VMAT to further elucidate their clinical roles in thoracic oncology are warranted.

While advances in radiotherapy technology had substantially reduced radiation RP incidence and pulmonary impairment, severe RP continued to significantly compromise quality of life and survival. Consequently, radiation oncologists required validated risk‐stratification criteria to modify treatment approaches—either through adaptive planning or alternative modalities—when RP risk was clinically significant. Our analysis of grade ≥ 2 RP risk factors in HT patients identified key clinical predictors: early hematological changes (≤1 month post‐radiotherapy) of the increasing white blood cells, neutrophils percentages of neutrophils and decreasing lymphocytes, percentage of lymphocytes; serum lactate increasing in 3 months post‐radiotherapy. The lymphopenia correlation (consistent with our prior retrospective findings^29^) may reflect radiation‐induced lymphocyte redistribution. Elevated lactate suggests hypoperfusion‐related hypoxia, with subclinical hypoxia often preceding radiological inflammation.^30^ This supports post‐radiotherapy lactate monitoring as a potential strategy for early RP detection and intervention. Dosimetrically, we found that bilateral lung V5, V20, and mean dose correlated with grade ≥2 RP, while RTOG 0617 found no V5 association with grade ≥3 RP,^8^ and our results align with other IMRT studies.^6^, ^7^ Furthermore, regarding immunotherapy timing and the risk of RP, Peri‐radiotherapy immune checkpoint inhibitors (ICIs) show variable RP risk profiles. PACIFIC trial reported no increased grade ≥ 3 RP with post‐chemoradiation durvalumab,^10^ and our study showed pre‐radiotherapy ICIs (≤3 months) did not elevate grade ≥2 RP risk, though large‐scale validation was warranted.

This study has limitations. Its single‐center design and sample size, though sufficient for the primary endpoint, limit the generalizability of the findings and precluded robust multivariate analysis of RP risk factors. Secondly, the absence of respiratory gating in the IMRT group may have introduced a confounding factor, a consideration for future larger‐scale studies. Finally, the use of different treatment planning systems for each arm may inherently introduce differences in dose distributions due to variations in optimization algorithms and strategies.

## AUTHOR CONTRIBUTIONS

Anmei Zhang, Yang Zhang, and Jingyun Yang were major contributors in writing the manuscript. Lu Chen and Na Wu carried out the data analyses, performed the statistical analysis. Jindong Qian, Hongya Dai, Dingqiang Yang, and Lirong Zhao helped with data collection. Liangzhi Zhong, Tianxiang Cui, Fan Yang, Wen Luo, Guangpeng Chen, and Yixing Gao performed the follow‐up; Guanghui Li designed, coordinated, and supervised the study and critically reviewed and discussed the manuscript.

## CONFLICT OF INTEREST STATEMENT

The authors declare no conflicts of interests.

## Supporting information



Supporting Information

## Data Availability

Data will be made available on request.
