# Supplementary material for: A controlled trial comparing dosimetry and radiation pneumonitis between tomotherapy and IMRT in patients with lung or esophageal cancer
Source: J Appl Clin Med Phys. 2026 Mar 18;27(3):e70537. doi: 10.1002/acm2.70537 (PMC13093822; doi:10.1002/acm2.70537)
Supplement: Supplementary file 1 — Supporting Information [file ACM2-27-e70537-s001.docx]

Supplementary Table S1: Incidence of radiation pneumonitis in subgroup analysis

|  | **lung cancer (n=69)** | | **P value** | | **esophageal cancer (n=41)** | | | | **P value** |
| --- | --- | --- | --- | --- | --- | --- | --- | --- | --- |
|  | IMRT **(n=35)** | HT **(n=34)** | |  | | IMRT **(n=21)** | HT **(n=20)** |  | |
| **All grade of RP** |  |  | | 0.54 | |  |  | 0.92 | |
| **Yes** | 27 (77.1%) | 24 (70.6%) | |  | | 15 (71.4%) | 14 (70.0%) |  | |
| **No** | 8 (22.9%) | 10 (29.4%) | |  | | 6 (28.6%) | 6 (30.0%) |  | |
| **≥ grade 2 RP** |  |  | | 0.56 | |  |  | 1.00 | |
| **Yes** | 7 (20.0%) | 5 (14.7%) | |  | | 2 (9.5%) | 2 (10.0%) |  | |
| **No** | 28 (80.0%) | 29 (85.3%) | |  | | 19 ( 90.5%) | 18 (90.0%) |  | |

RP=radiation pneumonitis

Data are n (%) or mean (SD) unless otherwise stated.
